# Islamic Perspectives on Polygenic Testing and Selection of IVF Embryos (PGT-P) for Optimal Intelligence and Other Non–Disease-Related Socially Desirable Traits

**DOI:** 10.1007/s11673-023-10293-0

**Published:** 2023-12-04

**Authors:** A. H. B. Chin, Q. Al-Balas, M. F. Ahmad, N. Alsomali, M. Ghaly

**Affiliations:** 1 Singapore Fertility and IVF Consultancy Pvt Ltd., Hong Lim Complex, 531A Upper Cross Street, Chinatown, Singapore; 2 Department of Medicinal Chemistry and Pharmacognosy, Faculty of Pharmacy, Jordan University of Science and Technology, Irbid, Jordan; 3 Advanced Reproductive Centre (ARC), Department of Obstetrics & Gynecology, Faculty of Medicine, Universiti Kebangsaan Malaysia, Jalan Yaacob Latif, Cheras, Kuala Lumpur, Malaysia; 4 Research Center, Neuroscience Research Department, King Fahad Medical City, Riyadh, Saudi Arabia; 5 Research Center for Islamic Legislation and Ethics (CILE), College of Islamic Studies, Hamad Bin Khalifa University, Ar-Rayyan, Qatar

**Keywords:** Fatwa, Islam, Muslims, Polygenic Risk Scores, Preimplantation genetic testing

## Abstract

In recent years, the genetic testing and selection of IVF embryos, known as preimplantation genetic testing (PGT), has gained much traction in clinical assisted reproduction for preventing transmission of genetic defects. However, a more recent ethically and morally controversial development in PGT is its possible use in selecting IVF embryos for optimal intelligence quotient (IQ) and other non–disease-related socially desirable traits, such as tallness, fair complexion, athletic ability, and eye and hair colour, based on polygenic risk scores (PRS), in what is referred to as PGT-P. Artificial intelligence (AI) and machine learning–based analysis of big data sets collated from genome sequencing of specific human ethnic populations can be used to estimate an individual embryo’s likelihood of developing such multifactorial traits by analysing the combination of specific genetic variants within its genome. Superficially, this technique appears compliant with Islamic principles and ethics. Because there is no modification of the human genome, there is no tampering with Allah’s creation (*taghyīr khalq Allah*). Nevertheless, a more critical analysis based on the five maxims of Islamic jurisprudence (*qawa'id fiqhiyyah*) that are often utilized in discourses on Islamic bioethics, namely *qaṣd* (intention), *yaqın̄* (certainty), *ḍarar* (injury), *ḍarūra* (necessity), and *`urf* (custom), would instead reveal some major ethical and moral flaws of this new medical technology in the selection of non–disease-related socially desirable traits, and its non-compliance with the spirit and essence of Islamic law (shariah). Muslim scholars, jurists, doctors, and biomedical scientists should debate this further and issue a fatwa on this new medical technology platform.

## Introduction

In recent years, the genetic testing and selection of IVF (in vitro fertilization) embryos, known as preimplantation genetic testing (PGT), has gained much traction in clinical assisted reproduction, for preventing the transmission of genetic defects to offspring (Fesahat, et al. 2020). A more controversial development is predictive genetic testing to select IVF embryos for optimal intelligence and other non–disease-related socially desirable traits such as tallness, fair complexion, athletic ability, and eye and hair colour, in what is referred to as preimplantation genetic testing for polygenic risks (PGT-P) (Chin 2022; Forzano, et al. 2022; Lencz, et al. 2022; Pagnaer, et al. 2021).

Because intelligence and other socially desirable traits are complex characteristics determined by the combination of multiple genes, polygenic scores are used to estimate an individual embryo’s likelihood of developing an adult-onset, multifactorial trait by analysing the combination of specific genetic variants within its genome (Chin 2022; Forzano, et al. 2022; Hujoel, et al. 2022; Lencz, et al. 2022; Pagnaer, et al. 2021). Unlike genome editing with CRISPR-Cas9 technology (Davies 2019), there are minimal risks because there is no genetic manipulation or modification. It is basically a technique for picking the “winning ticket” in the “genetic lottery” for optimal intelligence and other socially desirable traits among the various possible combination and permutation of genes that can occur during fertilization of the egg with sperm (Chin 2022; Forzano, et al. 2022; Hujoel, et al. 2022; Lencz, et al. 2022; Pagnaer, et al. 2021).

A recent large-scale survey conducted in the United States revealed that the desire to use polygenic testing to genetically enhance the intelligence and academic performance of one’s offspring is not confined to idiosyncratic and fringe elements within society (Heng 2023; Meyer, et al. 2023). Out of more than six thousand people polled, 38 per cent of respondents indicated that they would be willing to use polygenic testing to improve the chances of their child entering an elite university, while 28 per cent of respondents indicated they would even be willing to use gene editing to achieve this objective (Heng 2023; Meyer, et al. 2023). Hence, the utilization of PGT-P to screen and select IVF embryos for optimal intelligence and other non–disease-related socially desirable traits could likely represent lucrative business opportunities for IVF clinics worldwide. The question that arises is whether such a new technology application is religiously permissible for Muslims, who constitute a large fraction of the world’s population.

To start with, it must be noted that Islam in principle supports science and scientific inquiry, as part of its overall endorsement and encouragement for all beneficial knowledge (*ʿilm nāfi’*) (Abbott, et al. 2006; Khaled 2009), as attested by several verses in the Quran, such as verse 113 of *surah al-Nisa',* “And Allah has revealed to you the book and wisdom and has taught you that which you did not know. And ever has the favour of Allah upon you been great.” In addition, one of the Islamic principles favours having a strong and healthy population in term of morals, as well as physical and intellectual prowess (Mohammad 2006a), as implied by verse 26 of *Surah al-Qasas* in the Quran, “One of the women said, ‘O my father, hire him.’ Indeed, the best one you can hire is the strong and the trustworthy.”

## Overview of Islamic Laws (Shariah) and Jurisprudence (*Fiqh*)

The framework of Islamic jurisprudence (*fiqh*) constitutes robust and systematic methodologies for evaluating new medical techniques (Chin, et al. 2023; Mohammad 2020) such as PGT-P based on established Islamic laws (shariah). This includes *qawāʿid fiqhiyyah* (grand maxims of Islamic jurisprudence) (Mohammed 2005), *maqāṣid-al-shariah* (objectives of Islamic law) (Ibrahim, et al. 2019), and *maṣlaḥah-mafsadah* (benefit-harm risk assessment) (Chin, et al. 2023; Ibrahim, et al. 2019). *Qawāʿid fiqhiyyah* can be thought of as the tool and methodology of Islamic jurisprudence (*fiqh*), while *maqāṣid al-shariah* defines the objectives and destination (Chin, et al. 2023; Mohammad 2007). Overlying these is the need for rigorous and comprehensive benefit-harm risk assessment via *maṣlaḥah-mafsadah* (Ahmed 1995).

The five grand maxims of Islamic jurisprudence (*qawāʿid fiqhiyyah*) (Mohammad 2006b) are comprised of (1) matters are judged by their objectives (intention), (2) certainty cannot be overruled by doubt, (3) hardship begets facility (necessity), (4) harm (injury) must be eliminated, and (5) custom is authoritative (Chin, et al. 2023; Muhsin 2022). These maxims, encompassing the principles of *qaṣd* (intention), *yaqın̄* (certainty), *ḍarar* (injury), *ḍarūrah* (necessity), and *ʿurf* (custom) respectively (Chin et al. 2023; Mohammad 2006b), are often used for evaluating novel issues (*ijtihād*) (Saed 2015) such as new medical technologies, which are not explicitly mentioned and discussed by the Quran and *hadiths* (prophetic traditions). *Maqāṣid al-shariah* (higher objectives of Islamic law) (Ashour 2011) refers to the framework/philosophy of the five universal legal objectives of protecting religion (*hifz al-dīn*), life (*hifz al-nafs*), progeny (*hifz al-nasl*), intellect (*hifz al-ʿaql*), and wealth (*hifz al-māl*) (Chin, et al. 2023; Auda 2008). *Maṣlaḥah* refers to “the attainment of benefits and the avoidance of harms” (Ibrahim, et al. 2019), while *mafsadah* refers to harms and means of avoiding them (Chin, et al. 2023). Any action contravening shariah law is considered *mafsadah* (Mohammad 2008).

## Evaluation of Whether PGT-P is Aligned with Islamic Principles

A quick and superficial examination may lead to the conclusion that there are no harms, from an Islamic perspective, arising from the use of the PGT-P technique, based on the argument that tampering with Allah’s creation (*taghyīr khalq Allah*) is prohibited (Kamal 2003) and that in this case there is no gene editing or modification of the human genome. The mainstream position adopted by the overwhelming majority of Muslim religious scholars is that changing the human genome is prohibited unless it is done for compelling medical reasons (Alsomali and Hussein, 2021). Muslims believe that Allah has created humans in the best possible form, as stated in *surah al-Tin* verse 4 in the Quran, “Surely, We created man in the best of molds,” and therefore it would be wrong to tamper with Allah’s creation (Serour, et al. 2022). Hence, the alteration of Allah’s creation is prohibited and it is deemed to be “Satan’s act,” as implied by the following Quranic verse, “…and surely I [Satan] will command them and they will change Allah’s creation. And whoever takes Satan as a guardian instead of Allah has certainly suffered a tremendous loss” (*Surah al-Nisā’*, verse 119) (Kamal 2003).

**Fig. 1:**
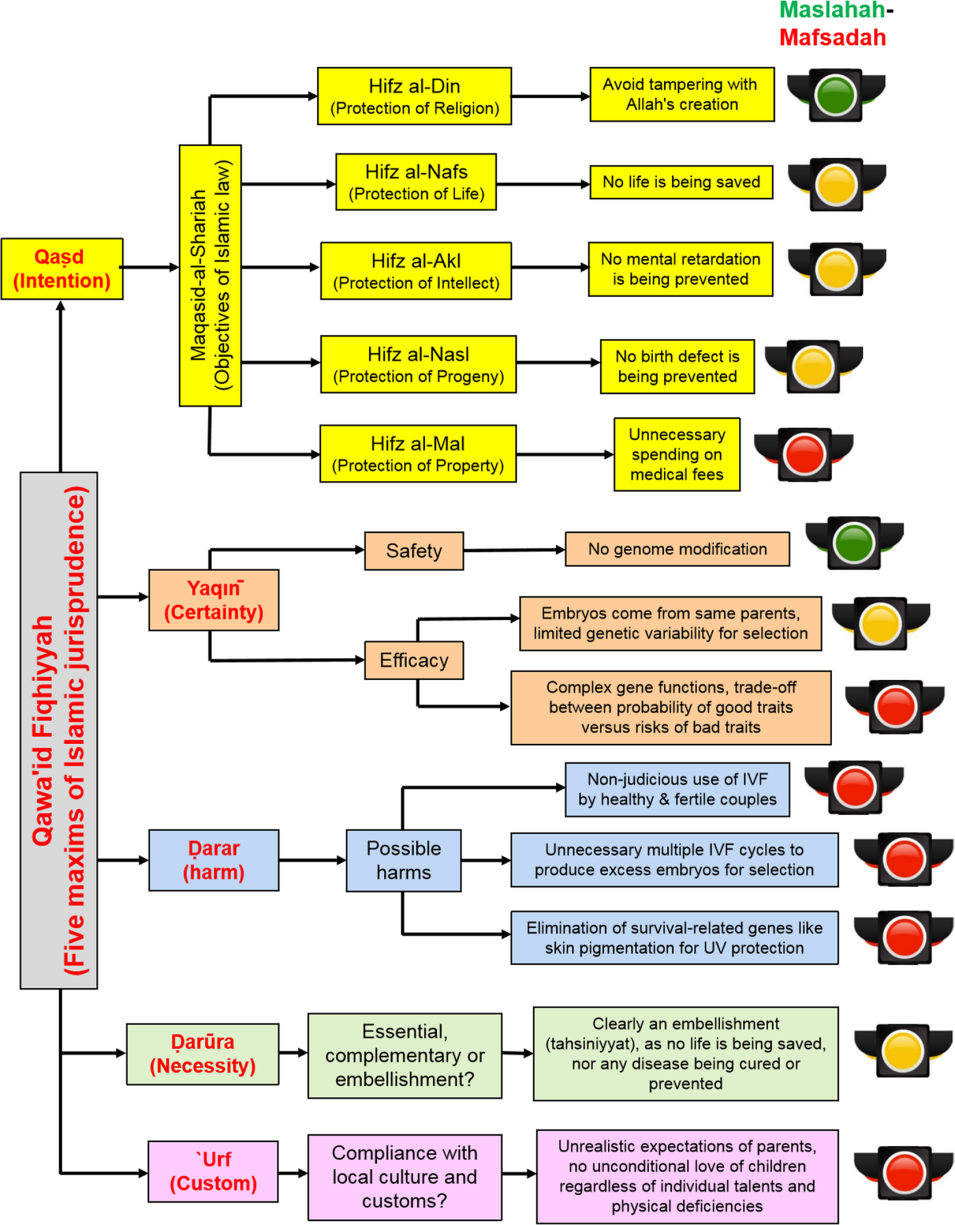
The morality and ethics of utilizing polygenic testing (PGT-P) to select for non–disease-related socially desirable traits in IVF embryos, based on *qawa’id fiqhiyyah. *The green traffic light represents “acceptable,” the yellow traffic light represents “caution,” while the red traffic light represents “unacceptable,” based on *maslahah-mafsadah* (public good versus detrimental effects on society)

However, a more critical analysis based on the five grand maxims of Islamic jurisprudence (*qawāʿid fiqhiyyah*) (Alsomali and Hussein, 2021; Mohammed 2005), which have been frequently employed in Islamic bioethical deliberations, may lead to a different conclusion. These five maxims revolve around the concepts of intention (*qaṣd*), certainty (*yaqın̄*), harm (*darar*), necessity (*darūra*), and custom (ʿ*urf*) (Mohammad 2006b). A serious engagement with these maxims and application of them to the case of using this new medical technology to select intelligence and other socially desirable traits would reveal major ethical and moral flaws and thus its incompatibility with the spirit and the essence of the Islamic religio-moral system (shariah) (figure 1).

## Alignment with the Maxim of *Qaṣd* (Intention)

As for the first maxim-related concept of intention (*qaṣd*), it holds that human acts (and by extension the use of such new medical technology) are to be judged by their goals and purposes (Chin, et al. 2023). In this case, the aim of PGT-P is neither to cure genetic diseases or to prevent transmitting them to the offspring. The purpose is rather to optimize the prospective child’s intelligence and other socially desirable traits. No disease is being cured, nor is any human life being saved. It is imperative to examine whether there is alignment with the five principal objectives of Islamic law (*maqāsid al-sharīʿah*) (Alsomali and Hussein, 2021; Ibrahim et al. 2019), which are meant to protect and safeguard religion (*dīn*), life (*nafs*), intellect (ʿ*aql*), offspring (*nasl*), and property (*māl*) (Chin, et al., 2023). Because the PGT-P technique avoids tampering with Allah’s creation (ʿ*abath*) (Kamal 2003) via the techniques of genome editing and genetic manipulation, the objective of preserving religion (*dīn*) seems to remain intact. However, the objectives of preserving life (*nafs*), intellect (ʿ*aql*), and offspring (*nasl*) will not be properly honoured, because no life is being saved, nor are mental retardation and birth defects being prevented. Hence, there may be unnecessary spending on expensive medical interventions, which would in turn contravene the objective of safeguarding property (*māl*) (Chin, et al. 2023). Whether or not spending on non-health or non-life-saving medical fees would be considered excessive is dependent on the socioeconomic conditions within the particular country or society where that Muslim community is based. For example, in an agrarian subsistence-level economy, such spending would be considered exorbitant and a flamboyant display of wealth, while in highly urbanized oil-rich states where clinical assisted reproduction procedures are commonplace due to state-subsidized healthcare systems, such spending may just be considered a trivial luxury.

Some proponents may argue that attempting to have quality offspring is encouraged in Islam, based on verse 74, *surah al-Furqan* of the Quran*,* which states that pious Muslims should pray to Allah in granting them children that would become the “apple of the eye” (*qurrat ʿayn*) (Ibrahim 1984). Although this could readily be interpreted as an intelligent and good-looking child, it must be noted that Islam in fact puts more emphasis on the righteousness and good moral character of the child, which is deemed to bring more benefits to the parents. Indeed, parents are believed to be rewarded for having a righteous child, as expounded by a *hadith* narrated by Abu Huraira that reported the prophet saying, “Verily, Allah Almighty will raise the status of his righteous servants in Paradise and they will say: O Lord, what is this? Allah will say: This is due to your child seeking forgiveness for you” (Mohammad 2014).

## Alignment with the Maxim of *Yaqın̄* (Certainty)

The maxim of certainty (*yaqın̄*) within the context of such novel bioethical issues refers to the current state of scientific knowledge and evidence of the safety and efficacy of new medical techniques (Chin, et al. 2023). As previously mentioned, there are minimal safety risks in the PGT-P technique because no genetic manipulation and genomic modification is involved. Nevertheless, there are limitations to the efficacy of PGT-P, based on certain technical constraints (Forzano, et al. 2022; Hujoel, et al. 2022; Lencz, et al. 2022; Pagnaer, et al. 2021). Because polygenic screening via PGT-P involves choosing between embryos with the same parents, this would substantially limit genetic variability and hence reduce its usefulness in selecting complex traits such as intelligence (Chin 2022). When screening for specific traits within such a small sample pool with little variability, there is a much-reduced number of possible outcomes, which obviates the usefulness of any selection algorithm (Chin 2022). Furthermore, many genes have multiple complex functions, which could be further complicated by various yet unknown interactions with other genes (Chin 2022). Very often, an increase in the probability of one “good” trait could lead to a corresponding increase in the risk of a “bad” trait (Chin 2022). For example, one study has reported genetic correlation between intelligence and risks of bipolar disorder (Smeland, et al. 2020). For any prospective parent, trading off risks between bipolar disorder and intelligence would certainly be daunting and difficult. However, when multiple trade-offs between other complex traits such as height, athletic ability, hair, and eye colour also need to be made, decision-making becomes extremely confusing and confounding for any prospective parent (Chin 2022).

Hence genetic testing companies may want to downplay such complex information and just present simplistic notions of “good” and “bad” genetic traits to prospective parents, lest they lose business when prospective parents become aware that they are facing such a confounding “paradox of choices” (Chin 2022). The lack of informed consent due to withholding such vital information from patients would thus be a violation of one of the key principles in the Islamic tradition, namely one’s authority to decide about one’s body on the basis of sufficient knowledge (Mohammad 2008). Exercising one’s free will on the basis of well-weighed judgement represents the foundation of one’s moral responsibility and accountability before Allah (Mohammad 2008). Indeed, the concept of patient consent in Islam is expounded by *surah al-Nisa'*, verse 4, which states “O you who have believed, do not consume one another’s wealth unjustly but only [in lawful] business by mutual consent.” Moreover, Islam places great emphasis on the concept of *amânah* (trustworthiness), which forms the basis of patient informed consent (Zulham 2014). Nevertheless, it must be noted that healthcare laws governing patient informed consent vary greatly in stringency from country to country, and in most Muslim-majority countries these would usually fall within the purview of civil legal code, rather than religious (shariah) laws.

## Alignment with the Maxim of *Darar* (Harm)

The maxim of harm (*darar*) raises the question of what possible harm could be caused by new medical techniques (Chin, et al. 2023). In the case of PGT-P that does not involve any genetic modification, potential harm does not arise from the technique itself but rather from its possible misapplication and abuse. For example, PGT-P might promote non-judicious use of IVF and other clinical assisted reproduction procedures that were originally developed and intended for infertility treatment (Chin 2022). In order to avail themselves of opportunities for embryo testing and selection to optimize intelligence and other socially desirable traits in their offspring, healthy and fertile couples might deliberately choose to unnecessarily undergo IVF (Chin 2022). Social and peer pressure may make such genetic testing irresistible to the rich and upper middle-classes, especially if this becomes trendy and fashionable among their friends, relatives, and colleagues. In particular, prospective parents might feel guilty about not giving their children the best start in life by not doing such expensive genetic testing via IVF, which is also highly invasive and risky. Hence, unnecessarily putting healthy and fertile patients to such gruelling medical procedures just for the sake of polygenic testing and selection of embryos via PGT-P must be considered “bad medicine” and clinical malpractice (Chin 2022). Nevertheless, due to variation in the medical professional code of conduct governing fertility doctors in different Muslim-majority countries, there is no standard consensus on whether it is ethically acceptable to allow healthy and fertile couples to undergo IVF procedures at their own volition.

Even for patients with genuine fertility problems, PGT-P would unduly pressure women to unnecessarily undergo multiple IVF cycles to produce excess embryos for polygenic testing and selection. This could add unnecessary stress and confusion to the already difficult and challenging IVF process and encourage patients to discard embryos unnecessarily, thus detracting from the main aim of conceiving a child in IVF treatment (Chin 2022).

More worryingly, large-scale commercialization of PGT-P would give the deceptive impression that the sought-after “good genes” and the need-to-be-avoided “bad genes” are classified on the basis of agreed-upon and fixed criteria, although this is not usually the case. Thus, the widespread application of this technology could lead to gradual elimination of what many would consider “bad genes,” although they had in fact ensured the survival of our distant ancestors in various different hostile environments over countless pestilences and plagues (Chin 2022). For example, there is scientific evidence that genetic predisposition to obesity and type II diabetes actually conferred survival advantages to our stone age ancestors in a food-scarce environment (Joffe and Zimmet 1998; Stöger 2008). What if future climate change results in droughts and famines? Would such “bad genes” that enable more efficient energy metabolism with reduced food intake now be considered “good genes” (Chin 2022)? Similarly, obsession with the beauty standard of fair skin might lead to elimination of pigmentation genes that could ensure our survival with more UV radiation entering our atmosphere (Sturm 1998), as the ozone layer gradually gets depleted. Hence, many so-called “bad genes” and “socially undesirable traits” could be an insurance policy for the future survival of humanity in a chaotic, uncertain, and ever-changing world, and it would be an utter folly to want to completely eliminate these through polygenic testing (Chin 2022).

## Alignment with the Maxim of *Darūra* (Necessity)

The maxim of necessity (*darūra*) questions the level of need to use a new medical technique and makes us wonder whether there are any other less controversial alternatives available (Chin, et al. 2023). Broadly speaking, the need to use a new medical technology can be classified, in descending order, into three levels, namely essentials (*darūriyyāt*), complementary (*hājiyyāt*), and embellishments (*taḥsiniyyāt*) (Chin, et al. 2023). The general rule holds that the lower the level of need, the stricter the conditions will be to permit the use of such ethically controversial technologies. In the case of PGT-P for the selection of optimal intelligence and other socially desirable traits, no disease is being cured nor any life being saved. Hence, it would fall into the category of embellishments (*tahsiniyyat*).

## Alignment with the Maxim of *‘Urf* (Custom)

The maxim of custom (*ʿurf*) refers to taking local customs and norms into consideration when religio-moral judgments are being made concerning novel issue such as new medical techniques (Chin, et al. 2023). Certainly, different Muslim communities with varying local cultures and socioeconomic conditions will place different emphasis and priorities on socially desirable traits such as intelligence and beauty standards linked to tallness and fair complexion (Heng 2023). Nevertheless, PGT-P for the selection of such traits would appear to contradict the almost universal norm across different cultures (both Islamic and non-Islamic) that parents are expected to show unconditional love for their children, regardless of their individual talents, intellectual abilities, or physical conditions. There is a risk that after having spent so much money and effort, parents may have unrealistic expectations of their “specially selected” children, who may feel that they are being treated like a “lab rat” in a scientific experiment and that their parents fail to love them unconditionally as who they are (Chin 2022). This would not only be an affront to universal family values but also to basic human dignity. Moreover, there are also social justice and inequality issues pertaining to utilization of PGT-P for selection of non–disease-related socially desirable traits to have genetically advantaged offspring, because only the rich will be able to access such expensive technologies. Some bioethicists have even posited that over several generations of cumulative genetic enhancement, this may lead to permanent stratification of mankind into two classes of genetically advantaged “haves” and “have-nots” (Pfeffier-Billaeuer 2022) which could in turn result in new forms of slavery and exploitation. Muslim communities will definitely view and perceive such issues differently from the perspective of their own local culture and varying state of socioeconomic development.

## Conclusion

In conclusion, upon closer inspection and critical analysis based on the grand maxims of Islamic jurisprudence (*qawāʿid fiqhiyyah*) (Mohammed 2005), higher objectives of Shariah (*maqāṣid-al-sharīʿah*) (Ibrahim, et al. 2019), and the religio-culturally harm-benefit (*maslahah-mafsadah*) assessment (Ibrahim, et al. 2019), the application of PGT-P in human clinical assisted reproduction for the selection of optimal intelligence and other socially desirable traits is in principle a highly contentious and controversial issue from an Islamic perspective. To develop detailed and nuanced positions on what to permit or prohibit in the use of this new technology, there is a need to initiate interdisciplinary bioethical discussions involving different stakeholders, especially among Muslim religious scholars, doctors, and biomedical scientists.
